# Hesperidin Protects Human HaCaT Keratinocytes from Particulate Matter 2.5-Induced Apoptosis via the Inhibition of Oxidative Stress and Autophagy

**DOI:** 10.3390/antiox11071363

**Published:** 2022-07-14

**Authors:** Pincha Devage Sameera Madushan Fernando, Mei Jing Piao, Kyoung Ah Kang, Ao Xuan Zhen, Herath Mudiyanselage Udari Lakmini Herath, Hee Kyoung Kang, Yung Hyun Choi, Jin Won Hyun

**Affiliations:** 1 Department of Biochemistry, College of Medicine, Jeju National University, Jeju 63243, Korea; sameera@stu.jejunu.ac.kr (P.D.S.M.F.); mjpiao@jejunu.ac.kr (M.J.P.); legna07@jejunu.ac.kr (K.A.K.); zhenaoxuan705@stu.jejunu.ac.kr (A.X.Z.); lakmini@stu.jejunu.ac.kr (H.M.U.L.H.); 2 Jeju Research Center for Natural Medicine, Jeju National University, Jeju 63243, Korea; pharmkhk@jejunu.ac.kr; 3 Department of Pharmacology, College of Medicine, Jeju National University, Jeju 63243, Korea; 4 Department of Biochemistry, Dongeui University College of Korean Medicine, Busan 47227, Korea; choiyh@deu.ac.kr

**Keywords:** hesperidin, particulate matter 2.5, human keratinocyte, autophagy, apoptosis, mitogen-activated protein kinase

## Abstract

Numerous epidemiological studies have reported that particulate matter 2.5 (PM_2.5_) causes skin aging and skin inflammation and impairs skin homeostasis. Hesperidin, a bioflavonoid that is abundant in citrus species, reportedly has anti-inflammatory properties. In this study, we evaluated the cytoprotective effect of hesperidin against PM_2.5_-mediated damage in a human skin cell line (HaCaT). Hesperidin reduced PM_2.5_-induced intracellular reactive oxygen species (ROS) generation and oxidative cellular/organelle damage. PM_2.5_ increased the proportion of acridine orange-positive cells, levels of autophagy-related proteins, beclin-1 and microtubule-associated protein light chain 3, and apoptosis-related proteins, B-cell lymphoma-2-associated X protein, cleaved caspase-3, and cleaved caspase-9. However, hesperidin ameliorated PM_2.5_-induced autophagy and apoptosis. PM_2.5_ promoted cellular apoptosis via mitogen-activated protein kinase (MAPK) activation by promoting the phosphorylation of extracellular signal-regulated kinase, c-Jun N-terminal kinase, and p38. The MAPK inhibitors U0126, SP600125, and SB203580 along with hesperidin exerted a protective effect against PM_2.5_-induced cellular apoptosis. Furthermore, hesperidin restored PM_2.5_-mediated reduction in cell viability via Akt activation; this was also confirmed using LY294002 (a phosphoinositide 3-kinase inhibitor). Overall, hesperidin shows therapeutic potential against PM_2.5_-induced skin damage by mitigating excessive ROS accumulation, autophagy, and apoptosis.

## 1. Introduction

Hesperidin, a major bioflavonoid, is abundant in citrus species such as sweet oranges and tangerines, especially in their peels [1]. Hesperidin consists of aglycone, hesperetin, and the sugar rutinoside [2]. Both hesperidin and hesperetin exhibit various biological activities including anti-inflammatory, antibacterial, and antitumor properties, as well as the potential to reduce capillary permeability [3,4]. Furthermore, hesperidin has been shown to have an inhibitory effect against SARS-CoV-2, the causative agent of COVID-19 [4]. 

Particulate matter (PM) exists in the atmosphere as a collection of liquid and solid particles. Depending on the source, PM has different chemical compositions and sizes, and PM with an aerodynamic diameter less than 2.5 µm is considered as PM_2.5_ [5]. PM is classified into two classes: primary particles and secondary particles. The primary particles are directly emitted from the source, such as fires, unpaved roads, and construction sites, whereas the secondary particles are formed through chemical reactions (automobiles, industrial zones, and power plants) [6]. The chemical composition of PM could be a composite of polycyclic aromatic hydrocarbons (naphthalene, acenaphthene, pyrene, benzo[k]fluoranthene, and benzo[g,h,i]perylene) and heavy metals (Fe, Zn, Pb, As, Cd, Cr, Cu, and Ni) [7,8]. The toxicity of PM depends on its source, chemical composition, and particle size [9,10]. Recently, we reported that PM_2.5_ causes skin damage, skin senescence, and skin inflammation by generating intracellular reactive oxygen species (ROS) [11,12,13]. 

The skin is the largest organ in the human body and is composed of multiple layers. The skin acts as a barrier between the body and the environment; is involved in thermoregulation; and protects the body from mechanical injuries, substances, and radiation [14]. There are several signaling pathways and mechanisms that maintain cellular homeostasis in the skin [15]. A range of extracellular harmful stimuli including PM may trigger cellular damage such as apoptosis, an irreversible event that alters cellular biochemical and morphological properties [16]. Therefore, in this study, we investigated the therapeutic potential of hesperidin against PM_2.5_-mediated cellular damage through the regulation of autophagy and apoptosis.

## 2. Materials and Methods

### 2.1. Reagents

Hesperidin (C_28_H_34_O_15_) was purchased from Sigma-Aldrich Inc. (St. Louis, MO, USA) and dissolved in dimethyl sulfoxide (DMSO). Standard diesel PM_2.5_ (SRM 1650b) issued by the National Institute of Standards and Technology (NIST, Gaithersburg, MD, USA) was purchased from Sigma-Aldrich Inc. and dissolved in DMSO. LY294002 (a phosphoinositide 3-kinase (PI3K) inhibitor), U0126 (a mitogen-activated protein kinase (MEK) inhibitor), SP600125 (a c-Jun N-terminal kinase (JNK) inhibitor), and SB203580 (a p38 inhibitor) were purchased from Calbiochem (San Diego, CA, USA) [17,18].

### 2.2. Cell Culture

The human HaCaT keratinocyte cell line was obtained from Cell Lines Service (Heidelberg, Germany). The cells were cultured in Dulbecco’s Modified Eagle’s Medium (DMEM) supplemented with 10% fetal bovine serum and antibiotic-antimycotic (100 units/mL penicillin, 100 µg/mL streptomycin, and 0.25 µg/mL amphotericin B). The cultures were maintained in a humidified incubator at 37 °C with 5% CO_2_ [19]. 

### 2.3. Intracellular ROS Detection 

2′,7′-Dichlorodihydrofluorescein diacetate H_2_DCFDA (Molecular Probes, Eugene, OR, USA) was used to measure intracellular ROS levels. The cells were cultured at a density of 1.5 × 10^5^ cells/mL in a six-well plate and incubated for 16 h. Thereafter, each corresponding well was treated with hesperidin (50 µM) and PM_2.5_ (50 µg/mL). Intracellular ROS levels were assessed using a flow cytometer (Becton Dickinson, Franklin Lakes, NJ, USA) after staining the cells with H_2_DCFDA. The cells were seeded (1 × 10^5^ cells/mL) on a chamber slide and incubated for 16 h, after which hesperidin and PM_2.5_ were added. Then, the cells were stained with H_2_DCFDA, and images were captured using a confocal microscope (Carl Zeiss, Oberkochen, Germany). 

### 2.4. Cell Viability Assessment

The colorimetric 3-(4,5-dimethylthiazol-2-yl)-2,5-diphenyltetrazolium bromide (MTT; Amresco Inc., Cleveland, OH, USA) assay was used to quantify cell viability. The cells were seeded at a density of 1.0 × 10^5^ cells/well in a 24-well plate and incubated for 16 h at 37 °C in a humidified atmosphere with 5% CO_2_. The cells were then treated with hesperidin (50 µM), PM_2.5_ (50 µg/mL), bafilomycin A1 (BAF, 10 nM; Sigma-Aldrich), LY294002 (50 µM), U0126 (50 nM), SP600125 (5 µM), and SB203580 (10 µM) based on each representative treatment group. Thereafter, 100 µL of MTT (2 mg/mL) was added to each well and incubated for 4 h to optimize the formation of formazan crystals. The formazan crystals were dissolved in DMSO, and the absorbance of the samples was determined using a scanning multi-well spectrophotometer (Sunrise; Tecan, Männedorf, Switzerland) at a wavelength of 540 nm. The cell viability was further confirmed using the trypan blue assay. Briefly, the cells were cultured in a six-well plate at a density of 0.8 × 10^5^ cells/mL, and then incubated for 16 h. The incubated cells were treated with hesperidin and PM_2.5_ for 24 h. Thereafter, dead cells were stained with 0.1% trypan blue solution. Cell viability was calculated as follows: live cells/(live cells + dead cells) × 100 [19]. 

### 2.5. Lipid Peroxidation Assay

The cells were cultured at a density of 1.0 × 10^5^ cells/mL in a four-well glass chamber slide. After 16 h of incubation, the cells were treated with hesperidin (50 µM) and PM_2.5_ (50 µg/mL), and then incubated for another 24 h. After staining with diphenyl-1-pyrenylphosphine (DPPP) (Molecular Probes), lipid adducts of DPPP oxide were detected via confocal microscopy.

### 2.6. Protein Carbonylation Assay

The cells were cultured at a density of 1.5 × 10^5^ cells/mL and incubated for 16 h. Thereafter, the cells were treated with hesperidin (50 µM) and PM_2.5_ (50 µg/mL), and then incubated for another 24 h. All treated cells were harvested and protein oxidation was detected using the Oxiselect™ protein carbonyl ELISA kit (Cell Biolabs, San Diego, CA, USA) following the manufacturer’s instructions.

### 2.7. Single-Cell Gel Electrophoresis Assay (Comet Assay)

The cells were treated with hesperidin (50 µM) and PM_2.5_ (50 µg/mL) for 30 min in microtubes based on each representative treatment group. The microtubes were then centrifuged to collect cells, which were fixed on glass slides by suspending in 1% low-melting-point agarose. After fixation, the slides were dipped for 1 h in a lysis buffer of 10 mM Tris-HCl (pH 10), containing NaCl (2.5 M), Na_2_EDTA (100 mM), Triton X-100 (1%), and DMSO (10%). The lysed cells on the slides were subjected to electrophoresis (25 V and 300 mA) for 20 min. Finally, the cells on the slides were stained with 1% ethidium bromide, and the comet tail length was measured using a fluorescence microscope equipped with image analysis software (Kinetic Imaging, Komet 5.5; Andor, Oxford, UK) [11].

### 2.8. Detection of 8-Oxoguanine (8-oxoG) 

The cells were cultured in a four-well chamber slide at a density of 1.5 × 10^5^ cells/mL and incubated for 16 h and treated with hesperidin (50 µM) and/or PM_2.5_ (50 µg/mL) for another 24 h. The expression of 8-oxoG (an oxidative DNA damage indicator) was detected via avidin-tetramethylrhodamine isothiocyanate (TRITC) conjugate (Sigma-Aldrich) staining. Images were captured using a confocal microscope.

### 2.9. Quantification of Cellular Ca^2+^ Level

The cells were cultured at a density of 1 × 10^5^ cell/mL in a four-well glass chamber slide and incubated for 16 h at 37 °C with 5% CO_2_. Thereafter, the cells were treated with hesperidin (50 µM) and/or PM_2.5_ (50 µg/mL) for 24 h. The treated cells were stained with Fluo-4-AM (Molecular Probes), and images were captured using a confocal microscope.

### 2.10. Mitochondrial Membrane Potential (Δψ_m_) Analysis

The cells were cultured at a density of 1 × 10^5^ cell/mL in a 4-well glass chamber slide and incubated for 16 h at 37 °C with 5% CO_2_ in a humidified atmosphere. The cells were then treated with hesperidin (50 µM) and exposed to PM_2.5_ (50 µg/mL) for 24 h. The cells were stained with 5,5′,6,6′-tetrachloro-1,1′,3,3′-tetraethylbenzimidazolylcarbocyanine iodide (JC-1; Invitrogen, Carlsbad, CA, USA) and analyzed using confocal microscopy.

### 2.11. Acridine Orange Morphology Assessment

Hesperidin (50 µM) and/or PM_2.5_ (50 µg/mL)-treated cells were treated with acridine orange (5 µM; Invitrogen) and incubated for 15 min to assess autophagy. Fluorescence was detected using a fluorescence microscope (BH2-RFL-T3; Olympus, Tokyo, Japan).

### 2.12. Western Blotting

After each treatment with hesperidin (50 µM) and PM_2.5_ (50 µg/mL), the cells were subjected to total protein extraction using PRO-PREP™ protein extraction solution (iNtRON Biotechnology, Seoul, Korea). The collected lysates were centrifuged at 13,000 rpm for 5 min. The extracted cell lysates were used to quantify protein levels using a protein assay reagent kit (Bio-Rad, Hercules, CA, USA). Resolving gel sheets (12% SDS-PAGE) were used to separate the proteins based on their molecular mass. The separated proteins were transferred from the gel sheets to nitrocellulose membranes. The nitrocellulose membranes were blocked with 3% bovine serum albumin for 1 h at 20 °C. The membranes were then incubated with the corresponding primary antibodies at 4 °C overnight. Primary antibodies were used to detect beclin-1, microtubule-associated protein-light chain 3 (LC3), caspase-9, caspase-3, JNK, phospho-JNK, Akt, phospho-Akt, phospho-p38 (Cell Signaling Technology, Beverly, MA, USA), Bcl-associated X protein (Bax), B-cell lymphoma-2 (Bcl-2), extracellular signal-regulated kinase (ERK), phospho-ERK, p38, and actin (Santa Cruz Biotechnology, Santa Cruz, CA, USA). After incubation with the primary antibodies, the membranes were incubated with the relevant secondary antibodies for another 1 h at room temperature. Subsequently, the membranes were treated with Amersham ECL western blotting detection reagent (GE Healthcare, Buckinghamshire, UK), and exposed to X-ray films (Agfa NV, Mortsel, Belgium) to visualize the protein bands. Bax, LC3, phospho-ERK, and phospho-p38 antibodies were monoclonal and the other antibodies were polyclonal [17,20,21].

### 2.13. Hoechst 33342 Staining 

The DNA-specific fluorescent dye Hoechst 33342 (Sigma-Aldrich) was used to detect apoptosis. The cells were seeded in a 24-well plate at a density of 1 × 10^5^ cells/mL and incubated for 16 h. The cells were then treated with hesperidin (50 µM) and/or PM_2.5_ (50 µg/mL), BAF (10 nM), U0126 (50 nM), SB203580 (10 µM), and SP600125 (5 µM). After each treatment, the cells were stained with Hoechst 33342 (20 µM) and visualized using a fluorescence microscope equipped with a Cool SNAP-Pro color digital camera (Media Cybernetics, Silver Spring, MD, USA) [21].

### 2.14. Statistical Analysis

All experiments were performed in triplicate. Data are presented as mean ± standard error and were analyzed using Tukey’s test and analysis of variance, using Sigma Stat 3.5 (Systat Software Inc., San Jose, CA, USA). Results with *p* < 0.05 were considered statistically significant.

## 3. Results

### 3.1. Hesperidin Restored PM_2.5_-Mediated Reduced Cell Viability by Mitigating Intracellular ROS Generation

**Figure 1:**
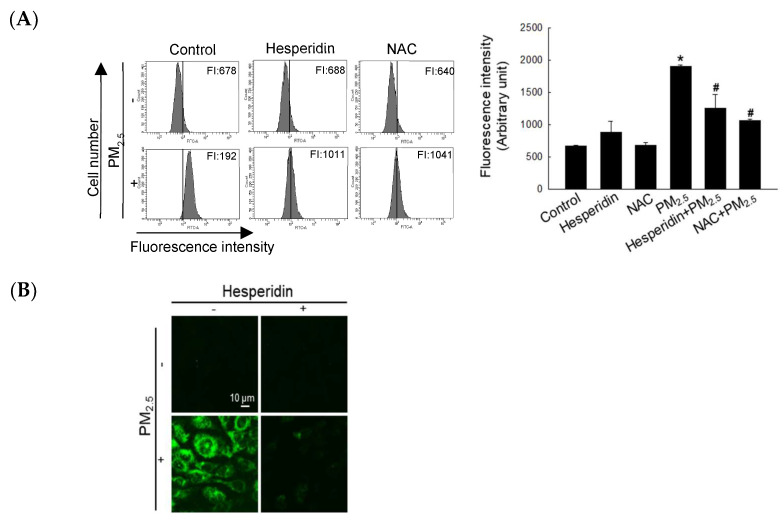

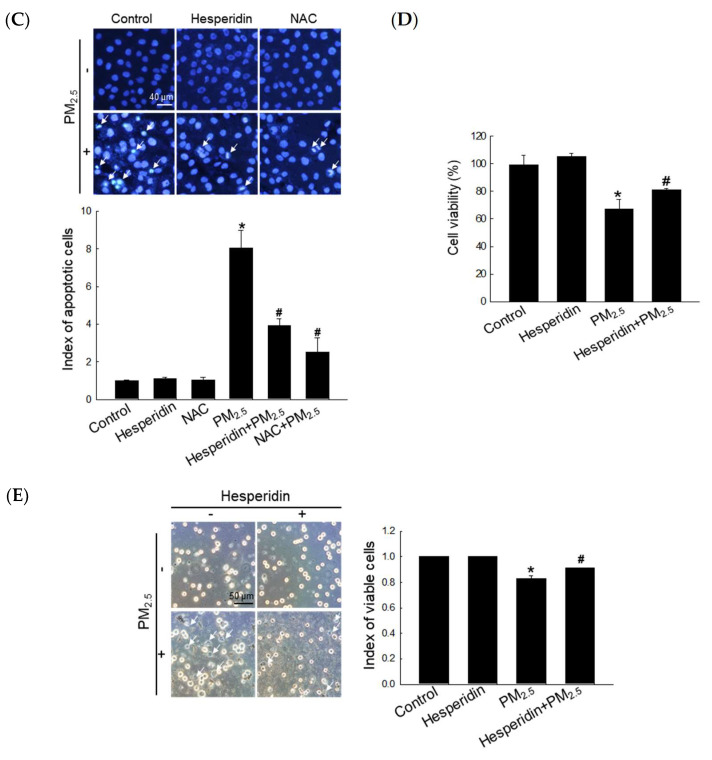
Hesperidin alleviated intracellular ROS generation and cell viability reduction. Cells were pretreated with hesperidin (50 µM) or NAC (1 mM) for 1 h, and then exposed to PM_2.5_ (50 µg/mL) for another 24 h. Intracellular ROS generation was observed using (**A**) flow cytometry and (**B**) confocal microscopy after H_2_DCFDA staining. (**C**) Apoptotic body formation was detected via Hoechst 33342 staining. Arrow indicates apoptotic body. (**D**) MTT and (**E**) trypan blue assays were used to determine cell viability. Arrows indicate the dead cells. * *p* < 0.05 and ^#^ *p* < 0.05 compared with the control cells and PM_2.5_-treated cells, respectively.

In our previous, we showed that 50 µM hesperidin exerted cytoprotective effects against UVB in human HaCaT keratinocytes [22]. Therefore, we selected 50 µM as the optimum concentration for further analysis. Intracellular ROS levels were quantified via flow cytometry of H_2_DCFDA staining and we compared the ROS scavenging ability of hesperidin with that of N-acetylcysteine (NAC, a well-known antioxidant). Both hesperidin- and NAC-treated groups showed a significant reduction in PM_2.5_-induced intracellular ROS levels (Figure 1A). As shown in Figure 1B, hesperidin also alleviated the PM_2.5_-induced intracellular ROS levels, which were assessed via the image of H_2_DCFDA staining. Thereafter, we assessed the effect of hesperidin against PM_2.5_-induced cellular apoptosis. The PM_2.5_-treated cells showed the highest apoptotic index, whereas the NAC- and hesperidin-treated cells showed a significant decrease (Figure 1C). As shown in Figure 1D, PM_2.5_ reduced cell viability (68%) compared with the control, as assessed using the MTT assay, whereas hesperidin restored 82% cell viability. This result was further confirmed using trypan blue staining, indicating that hesperidin has the potential to restore the PM_2.5_-mitigated cell viability index (Figure 1E). 

### 3.2. Hesperidin Inhibited the Damage of Cellular Components by PM_2.5_-Induced Oxidative Stress

**Figure 2:**
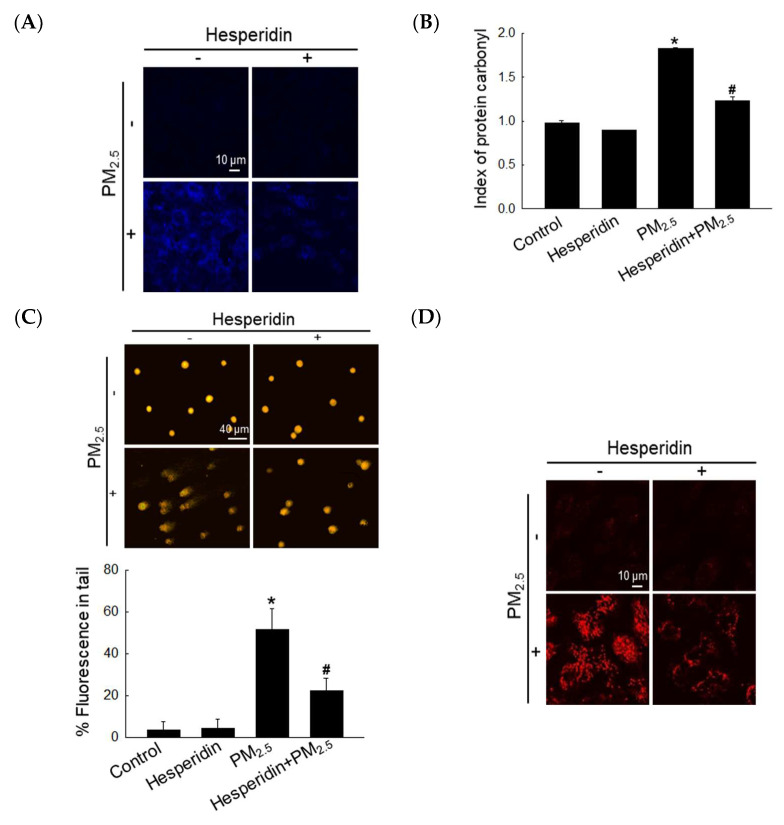
Hesperidin mitigated PM_2.5_-induced lipid peroxidation, protein carbonylation, and DNA damage. Cells were pretreated with hesperidin (50 µM) for 1 h, and then exposed to PM_2.5_ (50 µg/mL) for another 24 h. (**A**) Lipid peroxidation was detected via the image of DPPP staining. (**B**) Protein carbonylation was detected using the protein carbonyl ELISA kit. (**C**) Comet assay and (**D**) avidin-TRITC staining were used to assess DNA damage. * *p* < 0.05 and ^#^ *p* < 0.05 compared with the control cells and PM_2.5_-treated cells, respectively.

DPPP staining was used to assess the effect of hesperidin on PM_2.5_-induced lipid peroxidation [11]. As shown in Figure 2A, DPPP fluorescence intensity in the hesperidin-treated group was mitigated compared with that in the PM_2.5_-treated group. Protein carbonylation is considered a biomarker for oxidative stress due to protein damage [11]. PM_2.5_ triggered protein carbonylation, whereas hesperidin reversed it (Figure 2B). We then assessed the protective effect of hesperidin on PM_2.5_-induced DNA damage, which was confirmed using the comet assay (Figure 2C), where the hesperidin-treated group showed a significant reduction in comet tail length compared with the PM_2.5_-treated group. To further confirm the above result, we assessed the 8-oxoG level using avidin-TRITC staining; PM_2.5_ triggered the formation of 8-oxoG (high red fluorescence), whereas hesperidin reversed it (Figure 2D).

### 3.3. Hesperidin Mitigated PM_2.5_-Induced Mitochondrial Dysfunction and Autophagy Activation

**Figure 3:**
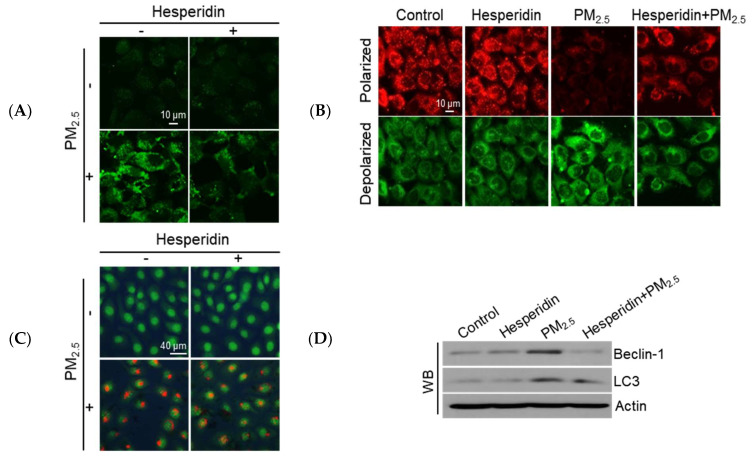
Hesperidin prevented PM_2.5_-induced intracellular Ca^2+^ accumulation, mitochondria dysfunction, and autophagy. Cells were pretreated with hesperidin (50 µM) for 1 h, and then exposed to PM_2.5_ (50 µg/mL) for another 24 h. (**A**) Intracellular Ca^2+^ level was detected using confocal microscopy after Fluo-4-AM staining. (**B**) The mitochondrial membrane potential was detected using confocal microscopy after JC-1 staining. (**C**) Autophagy was detected using fluorescence microscopy after acridine orange staining. (**D**) Cell lysates were subjected to western blotting of target proteins (beclin-1, LC3, and actin).

ROS can disrupt the cellular Ca^2+^ homeostasis, which could result in mitochondrial stress and autophagy induction [23,24]. PM_2.5_-induced oxidative stress resulted in both intracellular Ca^2+^ accumulation and mitochondrial damage [11]. As shown in Figure 3A, PM_2.5_ increased intracellular Ca^2+^ accumulation, which was alleviated by hesperidin. It has been reported that calcium overload in the cytoplasm enhances the opening of the mitochondrial permeability transition pores and results in mitochondrial membrane depolarization, impairs ATP production, and triggers cellular apoptosis [25]. We then assessed the mitochondrial membrane potential (Δψ_m_) via JC-1 staining, where red fluorescence represents the polarized status and green fluorescence represents the depolarized status. PM_2.5_ elevated mitochondrial depolarization, which was notably reversed by hesperidin (Figure 3B). Autophagy is triggered in response to mitochondrial depolarization to remove dysfunctional or damaged mitochondria [25]. We then assessed the effect of hesperidin on PM_2.5_-mediated autophagy activation using acridine orange, which detects autophagic lysosomes with orange/red fluorescence due to their intracellular acidity, which forms cytoplasmic vesicles [21]. As shown in Figure 3C, PM_2.5_ augmented the accumulation of intracellular vacuoles, which was mitigated by hesperidin. According to the western blotting results, PM_2.5_-treated cells prominently expressed autophagy-related proteins such as beclin-1 and LC3, whereas hesperidin mitigated the expression of these proteins (Figure 3D). 

### 3.4. Hesperidin Ameliorated Cellular Apoptosis via the Inhibition of PM_2.5_-Induced Autophagy

**Figure 4:**
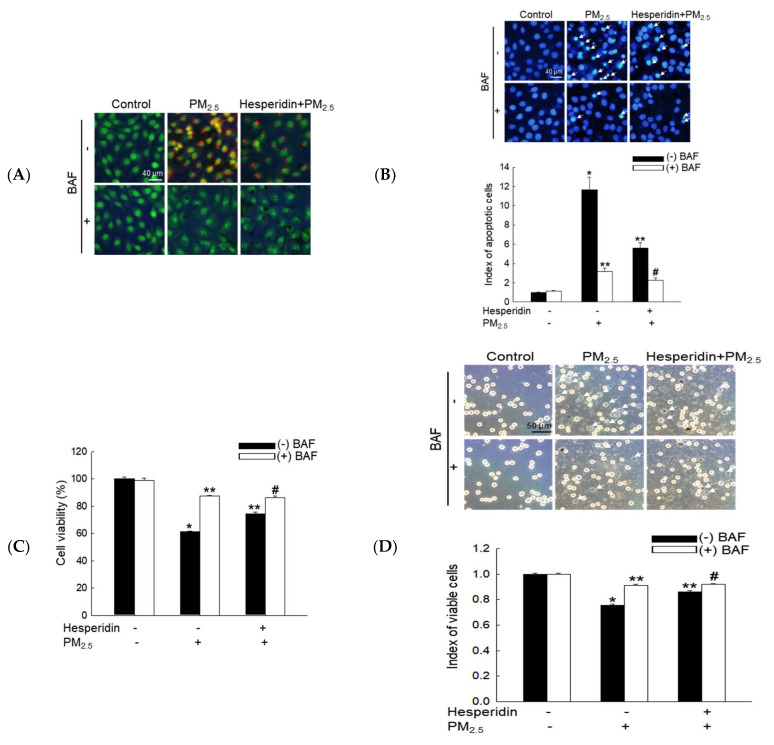
Hesperidin prevented cellular apoptosis and reduced cell viability via the inhibition of PM_2.5_-induced autophagy. Cells were pretreated with hesperidin (50 µM), BAF (10 nM), or both for 1 h, and then exposed to PM_2.5_ (50 µg/mL) for another 24 h. (**A**) Autophagy was detected using images captured via fluorescence microscopy after staining with acridine orange. (**B**) Apoptotic bodies were observed using Hoechst 33342 staining, and the arrows indicate apoptotic bodies. Cell viability was assessed via (**C**) MTT assay and (**D**) trypan blue staining, and the arrows indicate the dead cells. * *p* < 0.05, ** *p* < 0.05, and ^#^ *p* < 0.05 compared with BAF-untreated control cells, BAF-untreated PM_2.5_-exposed cells, and BAF-untreated hesperidin + PM_2.5_-exposed cells, respectively.

PM_2.5_-induced intracellular vacuole accumulation was further clarified using BAF, a lysosomal inhibitor [26]. Compared with BAF-treated cells, hesperidin-treated cells exhibited a considerable reduction in PM_2.5_-induced intracellular vacuole accumulation (Figure 4A). In addition, BAF, hesperidin, or both notably decreased PM_2.5_-induced apoptosis, as assessed using Hoechst 33342 staining (Figure 4B), resulting in the significant recovery of PM_2.5_-mediated reduced cell viability, as assessed using MTT and trypan blue assays (Figure 4C,D).

### 3.5. Hesperidin Inhibited PM_2.5_-Induced Cellular Apoptosis via MAPK Inactivation 

**Figure 5:**
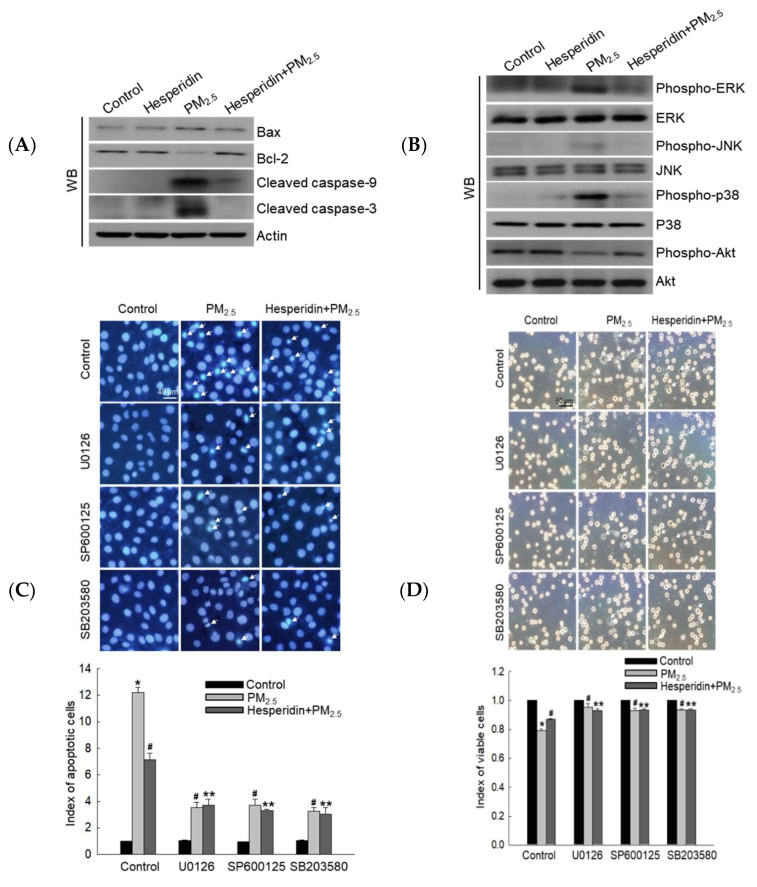

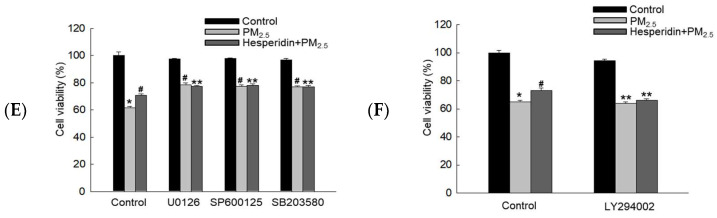
Hesperidin mitigated PM_2.5_-induced cell apoptosis and MAPK activation. Cells were pretreated with hesperidin (50 µM) for 1 h, and then exposed to PM_2.5_ (50 µg/mL) for another 24 h. (**A**) Cell lysates were subjected to western blotting for Bax, Bcl-2, cleaved caspase-9, cleaved caspase-3, (**B**) phospho-ERK, ERK, phospho-JNK, JNK, phospho-p38, p38, phospho-Akt, Akt. Actin was used as the loading control. Cells were pretreated with hesperidin (50 µM), U0126 (50 nM), SP600125 (5 µM), SB203580 (10 µM), and LY294002 (50 µM) for 1 h, and then exposed to PM_2.5_ (50 µg/mL) for 24 h. (**C**) Hoechst 33342 staining was used to assess cellular apoptosis, and the arrows indicate apoptotic bodies. Cell viability was assessed using (**D**) trypan blue, arrows indicating the dead cells, and (**E**,**F**) MTT assays. * *p* < 0.05, ^#^ *p* < 0.05, and ** *p* < 0.05 compared with the control cells, PM_2.5_-exposed cells, and hesperidin + PM_2.5_-exposed cells, respectively.

We assessed the expression of apoptosis-related proteins, Bax, Bcl-2, cleaved caspase-9, and cleaved caspase-3. PM_2.5_ enhanced the expression levels of Bax, cleaved caspase-9, and cleaved caspase-3 protein, but hesperidin reversed this pattern (Figure 5A). In addition, hesperidin increased the expression of anti-apoptotic protein Bcl-2, which was inhibited by PM_2.5_ exposure (Figure 5A). Considering the activation of the MAPK signaling pathway, we assessed the expression levels of the active form of intracellular ERK, JNK, p38, and Akt proteins. PM_2.5_-treated cells presented the upregulated expression of phospho-ERK, phospho-JNK, and phospho-p38, whereas hesperidin pretreatment mitigated phosphorylated protein expression (Figure 5B). Furthermore, hesperidin pretreatment upregulated the expression of phospho-Akt, which was reduced by PM_2.5_ exposure (Figure 5B). To confirm whether hesperidin exerted its cytoprotective effect against PM_2.5_ through the inhibition of MAPK pathway activation, we performed cellular apoptosis and cell viability assessments using MAPK pathway-related protein inhibitors. ERK, JNK, and p38 inhibitors decreased the PM_2.5_-induced apoptotic body formation, similar to the reduced pattern observed under hesperidin treatment (Figure 5C). To investigate the cell viability levels, the trypan blue and MTT assays were performed, and the results confirmed that all inhibitors increased PM_2.5_-mediated reduced cell viability, similar to the viability of hesperidin-treated cells (Figure 5D,E). Furthermore, to demonstrate that hesperidin antagonizes the repressive effect of PM_2.5_ on Akt activation, we performed the MTT assay with the PI3K inhibitor LY294002. The cell viability of the hesperidin + LY294002 + PM_2.5_ group decreased compared to that of the hesperidin + PM_2.5_ group, indicating that hesperidin has the potential to restore PM_2.5_-suppressed Akt via the activation of PI3K (Figure 5F).

## 4. Discussion

The present study showed that hesperidin alleviates the PM_2.5_-induced ROS generation and cellular macromolecule damage (DNA, protein, and lipid), thereby protecting against PM_2.5_-mediated cell viability reduction.

Autophagy involves the degradation of cellular organelles and recycling of damaged cellular organelles and abnormal proteins to maintain intracellular homeostasis. However, overactivation of autophagy may damage critical cellular components, resulting in aberrant cell shapes and cell death [27]. Excessive autophagy activation may contribute to apoptotic cell death via unchecked degradative processes [28]. Excessive ROS production can result in defective autophagy, hyperactivation, or inhibition of autophagic flux, and apoptosis [29]. In our previous study, we demonstrated that PM_2.5_ induced the generation of ROS and autophagy, resulting in apoptosis [11]. However, the present study showed that hesperidin alleviates both PM_2.5_-mediated autophagy activation and apoptosis via its antioxidant effect.

The MAPK pathway is activated in response to cellular stress, and it is implicated in cellular signaling that leads to cell death or survival [30]. Hesperidin mitigated PM_2.5_-mediated MAPK activation and cell apoptosis, which suggests that hesperidin exerts its therapeutic potential against PM_2.5_ via the inhibition of MAPK activation. It has been reported that the activation of PI3K and its downstream Akt could increase cell survival via the inhibition of apoptosis [18]. We observed that hesperidin could increase cell viability reduced by PM_2.5_ via Akt activation, suggesting that hesperidin has therapeutic potential against PM_2.5_-mediated cellular damage via the inhibition of MAPK activation as well as the activation of PI3K/Akt. It has been reported that PM_2.5_ could induce cell death in multiple ways including apoptosis, necrosis, pyroptosis, and ferroptosis [31,32]. For our further studies, we plan to evaluate the effects of hesperidin by focusing on PM_2.5_-induced apoptosis and other forms of cell death including ferroptosis and pyroptosis.

**Figure 6:**
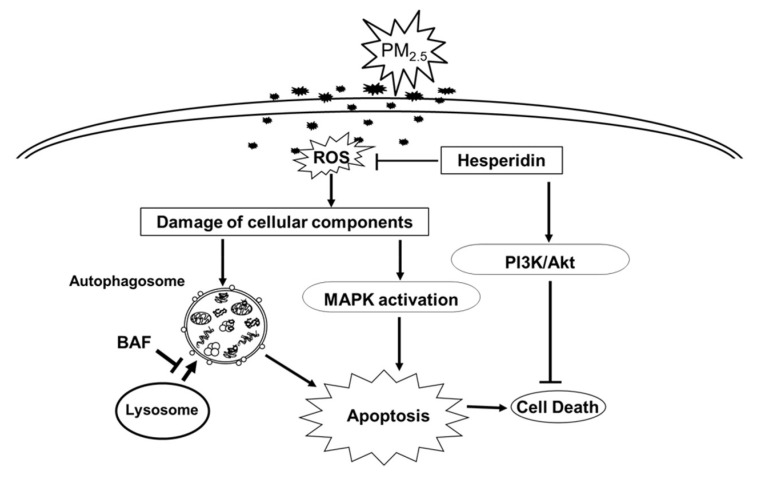
Schematic diagram summarizing the protective mechanism of hesperidin against PM_2.5_-induced cell damage. Hesperidin protects keratinocytes by suppressing PM_2.5_-induced intracellular ROS generation, intracellular macromolecules damage, autophagy activation, and cell apoptosis. Hesperidin alleviates cell apoptosis via the inhibition of PM_2.5_-induced MAPK activation as well as excessive autophagy activation, which may cause the uncontrolled degradation of intracellular components that eventually results in apoptosis and cell death. Furthermore, hesperidin restored the PM_2.5_-mediated decrease in cell viability via the activation of the PI3K/Akt pathway.

Overall, hesperidin mitigated PM_2.5_-induced macromolecular damage, alleviated autophagy hyperactivation, and inhibited cell apoptosis via the inhibition of MAPK activation, and induced cell survival via PI3K/Akt activation (Figure 6). 

## 5. Conclusions

Our study demonstrated that hesperidin exerts the protective effect against PM_2.5_-induced cell death by alleviating excessive ROS generation, macromolecule damage, excessive autophagy activation, and cell apoptosis. On the basis of our findings, hesperidin is a drug candidate for skin protection against air pollutants.

## Data Availability

Data is contained within the article.

## References

[1] Lahmer N., Belboukhari N., Cheriti A., Sekkoum K. (2015). Hesperidin and hesperitin preparation and purification from citrus sinensis peels. Der Pharma Chem..

[2] Ahmadi A., Shadboorestan A. (2015). Oxidative stress and cancer; the role of hesperidin, a citrus natural bioflavonoid, as a cancer chemoprotective agent. Nutr. Cancer.

[3] Iranshahi M., Rezaee R., Parhiz H., Roohbakhsh A., Soltani F. (2015). Protective effects of flavonoids against microbes and toxins: The cases of hesperidin and hesperetin. Life Sci..

[4] El Barky A.R., Mohamed T.M. (2020). Isolation, characterization and the biological activity of some natural components of marine sea cucumber and orange peel. Biomed. J. Sci. Tech. Res..

[5] Singh V., Singh S., Biswal A. (2021). Exceedances and trends of particulate matter (PM_2.5_) in five Indian megacities. Sci. Total Environ..

[6] Fiordelisi A., Piscitelli P., Trimarco B., Coscioni E., Iaccarino G., Sorriento D. (2017). The mechanisms of air pollution and particulate matter in cardiovascular diseases. Heart Fail. Rev..

[7] Janta R., Sekiguchi K., Yamaguchi R., Sopajaree K., Pongpiachan S., Chetiyanukornkul T. (2020). Ambient PM_2.5_, polycyclic aromatic hydrocarbons and biomass burning tracer in Mae Sot district, western Thailand. Atmos. Pollut. Res..

[8] Lei W., Zhang L., Xu J., Liu Z., Xin J., Li X., Zhao W. (2021). Spatiotemporal variations and source apportionment of metals in atmospheric particulate matter in Beijing and its surrounding areas. Atmos. Pollut. Res..

[9] Rönkkö T.J., Hirvonen M.R., Happo M.S., Ihantola T., Hakkarainen H., Martikainen M.V., Gu C., Wang Q.G., Jokiniemi J., Komppula M. (2021). Inflammatory responses of urban air PM modulated by chemical composition and different air quality situations in Nanjing, China. Environ. Res..

[10] Kumar P., Kalaiarasan G., Porter A.E., Pinna A., Kłosowski M.M., Demokritou P., Chung K.F., Pain C., Arvind D.K., Arcucci R. (2021). An overview of methods of fine and ultrafine particle collection for physicochemical characterisation and toxicity assessments. Sci. Total Environ..

[11] Piao M.J., Ahn M.J., Kang K.A., Ryu Y.S., Hyun Y.J., Shilnikova K., Zhen A.X., Jeong J.W., Choi Y.H., Kang H.K. (2018). Particulate matter 2.5 damages skin cells by inducing oxidative stress, subcellular organelle dysfunction, and apoptosis. Arch. Toxicol..

[12] Ryu Y.S., Kang K.A., Piao M.J., Ahn M.J., Yi J.M., Bossis G., Hyun Y.M., Park C.O., Hyun J.W. (2019). Particulate matter-induced senescence of skin keratinocytes involves oxidative stress-dependent epigenetic modifications. Exp. Mol. Med..

[13] Ryu Y.S., Kang K.A., Piao M.J., Ahn M.J., Yi J.M., Hyun Y.M., Kim S.H., Ko M.K., Park C.O., Hyun J.W. (2019). Particulate matter induces inflammatory cytokine production via activation of NFκB by TLR5-NOX4-ROS signaling in human skin keratinocyte and mouse skin. Redox Biol..

[14] Dąbrowska A.K., Spano F., Derler S., Adlhart C., Spencer N.D., Rossi R.M. (2018). The relationship between skin function, barrier properties, and body-dependent factors. Skin Res. Technol..

[15] Gomes A.P., Blenis J. (2015). A nexus for cellular homeostasis: The interplay between metabolic and signal transduction pathways. Curr. Opin. Biotechnol..

[16] Liu Y., Chen Y.Y., Cao J.Y., Tao F.B., Zhu X.X., Yao C.J., Chen D.J., Che Z., Zhao Q.H., Wen L.P. (2015). Oxidative stress, apoptosis, and cell cycle arrest are induced in primary fetal alveolar type II epithelial cells exposed to fine particulate matter from cooking oil fumes. Environ. Sci. Pollut. Res. Int..

[17] Ryu Y.S., Fernando P.D.S.M., Kang K.A., Piao M.J., Zhen A.X., Kang H.K., Koh Y.S., Hyun J.W. (2019). Marine compound 3-bromo-4, 5-dihydroxybenzaldehyde protects skin cells against oxidative damage via the Nrf2/HO-1 pathway. Mar. Drugs.

[18] Kang K.A., Wang Z.H., Zhang R., Piao M.J., Kim K.C., Kang S.S., Kim Y.W., Lee J., Park D., Hyun J.W. (2010). Myricetin protects cells against oxidative stress-induced apoptosis via regulation of PI3K/Akt and MAPK signaling pathways. Int. J. Mol. Sci..

[19] Zhen A.X., Hyun Y.J., Piao M.J., Fernando P.D.S.M., Kang K.A., Ahn M.J., Yi J.M., Kang H.K., Koh Y.S., Lee N.H. (2019). Eckol inhibits particulate matter 2.5-induced skin keratinocyte damage via MAPK signaling pathway. Mar. Drugs.

[20] Hyun Y.J., Piao M.J., Kang K.A., Zhen A.X., Fernando P.D.S.M., Kang H.K., Ahn Y.S., Hyun J.W. (2019). Effect of fermented fish oil on fine particulate matter-induced skin aging. Mar. Drugs.

[21] Zhen A.X., Piao M.J., Hyun Y.J., Kang K.A., Fernando P.D.S.M., Cho S.J., Ahn M.J., Hyun J.W. (2019). Diphlorethohydroxycarmalol attenuates fine particulate matter-induced subcellular skin dysfunction. Mar. Drugs.

[22] Hewage S.R.K.M., Piao M.J., Kang K.A., Ryu Y.S., Han X., Oh M.C., Jung U., Kim I.G., Hyun J.W. (2016). Hesperidin attenuates ultraviolet B-induced apoptosis by mitigating oxidative stress in human keratinocytes. Biomol. Ther..

[23] Görlach A., Bertram K., Hudecova S., Krizanova O. (2015). Calcium and ROS: A mutual interplay. Redox Biol..

[24] Yu K.N., Chang S.H., Park S.J., Lim J., Lee J., Yoon T.J., Kim J.S., Cho M.H. (2015). Titanium dioxide nanoparticles induce endoplasmic reticulum stress-mediated autophagic cell death via mitochondria-associated endoplasmic reticulum membrane disruption in normal lung cells. PLoS ONE.

[25] Lin D.S., Huang Y.W., Ho C.S., Hung P.L., Hsu M.H., Wang T.J., Wu T.Y., Lee T.H., Huang Z.D., Chang P.C. (2019). Oxidative insults and mitochondrial DNA mutation promote enhanced autophagy and mitophagy compromising cell viability in pluripotent cell model of mitochondrial disease. Cells.

[26] Park S.H., Park H.S., Lee J.H., Chi G.Y., Kim G.Y., Moon S.K., Chang Y.C., Hyun J.W., Kim W.J., Choi Y.H. (2013). Induction of endoplasmic reticulum stress-mediated apoptosis and non-canonical autophagy by luteolin in NCI-H460 lung carcinoma cells. Food Chem. Toxicol..

[27] Yi C., Si L., Xu J., Yang J., Wang Q., Wang X. (2020). Effect and mechanism of asiatic acid on autophagy in myocardial ischemia-reperfusion injury in vivo and in vitro. Exp. The. Med..

[28] Wang K. (2015). Autophagy and apoptosis in liver injury. Cell Cycle.

[29] Carresi C., Mollace R., Macrì R., Scicchitano M., Bosco F., Scarano F., Coppoletta A.R., Guarnieri L., Ruga S., Zito M.C. (2021). Oxidative stress triggers defective autophagy in endothelial cells: Role in atherothrombosis development. Antioxidants.

[30] Kang K.A., Piao M.J., Ryu Y.S., Hyun Y.J., Park J.E., Shilnikova K., Zhen A.X., Kang H.K., Koh Y.S., Jeong Y.J. (2017). Luteolin induces apoptotic cell death via antioxidant activity in human colon cancer cells. Int. J. Oncol..

[31] Hu R., Xie X.Y., Xu S.K., Wang Y.N., Jiang M., Wen L.R., Lai W., Guan L. (2017). PM_2.5_ exposure elicits oxidative stress responses and mitochondrial apoptosis pathway activation in HaCaT keratinocytes. Chin. Med. J..

[32] Wang Y., Zhong Y., Liao J., Wang G. (2021). PM_2.5_-related cell death patterns. Int. J. Med. Sci..

